# Direct Ink Write (DIW) 3D Printed Cellulose Nanocrystal Aerogel Structures

**DOI:** 10.1038/s41598-017-07771-y

**Published:** 2017-08-14

**Authors:** Vincent Chi-Fung Li, Conner K. Dunn, Zhe Zhang, Yulin Deng, H. Jerry Qi

**Affiliations:** 1 Renewable Bioproducts Institute at Georgia Institute of Technology, School of Chemical & Biomolecular Engineering, Atlanta, GA 30318 USA; 2 Renewable Bioproducts Institute at Georgia Institute of Technology, George W. Woodruff School of Mechanical Engineering, Atlanta, GA 30332 USA

## Abstract

Pure cellulose nanocrystal (CNC) aerogels with controlled 3D structures and inner pore architecture are printed using the direct ink write (DIW) technique. While traditional cellulosic aerogel processing approaches lack the ability to easily fabricate complete aerogel structures, DIW 3D printing followed by freeze drying can overcome this shortcoming and can produce CNC aerogels with minimal structural shrinkage or damage. The resultant products have great potential in applications such as tissue scaffold templates, drug delivery, packaging, etc., due to their inherent sustainability, biocompatibility, and biodegradability. Various 3D structures are successfully printed without support material, and the print quality can be improved with increasing CNC concentration and printing resolution. Dual pore CNC aerogel scaffolds are also successfully printed, where the customizable 3D structure and inner pore architecture can potentially enable advance CNC scaffold designs suited for specific cell integration requirements.

## Introduction

3D printing or additive manufacturing is revolutionizing the field of manufacturing, as the additive process can reduce the time needed to fabricate a highly customized object with complex geometry; the reduced fabrication time will be highly beneficial due to the ever increasing demands for customized products. Current 3D printers are reaching the ability to fabricate structures with micrometer or higher resolution, and 3D printing has already impacted manufacturing processes within industrial sectors such as aerospace, automotive, architecture, electronic, sensor engineering, dental, biomedical, and many others^1^. In fact, the 3D printing industry is expected to expand from 700 million dollars to 8.9 billion dollars within the next decade^2^.

There are now many 3D printing techniques for processing diverse materials. However, commonly used 3D printing materials, such as polyamide (PA) or acrylonitrile-butadiene-styrene (ABS)^3–5^, are petroleum based. Given the wide usage and declining supply of petrochemical resources, there is a high incentive for using more sustainable and renewable alternatives^6^. Cellulose nanocrystals (CNCs) have recently received significant attention due to their high Young’s modulus, high strength, light weight, low density, sustainability, biocompatibility, biodegradability, recyclability, and abundance nature^6–9^. As a result, it is conceivable and advantageous to substitute traditional 3D printing thermoplastics with cellulosic materials such as CNCs. In practical terms, wood pulp can be mechanically or chemically treated, and then acid hydrolysis can be performed to remove the amorphous regions^10, 11^. This leaves behind individual crystallites that are known as CNC. Depending on the cellulose source and processing conditions, the length and diameter of CNCs can respectively be around 100 to 500 nm and 10 to 30 nm^12–14^. They have intricate intra- and inter-cellulose chain hydrogen bonding that gives rise to a high axial elastic modulus of 110 to 220 GPa^7, 15^.

Although recent developments in processing highly functional 3D aerogel structures have been rapid^16, 17^, there is an increasing need for more biocompatible and biodegradable aerogels within the biomedical, cosmetics, pharmaceutical, and even packaging fields^18, 19^. As a result, there are increasing focus on processing purely cellulosic aerogels using microfibrillated cellulose (MFC)^20, 21^, cellulose nanofibrils (CNF)^10, 22–24^, or CNCs^25^. More commonly, the facile freeze drying approach is adopted because low density and high porosity aerogels can be obtained. Yang *et al*.^25^ fabricated chemically cross-linked CNC aerogels using aldehyde modified CNCs and hydrazide modified CNCs, which resulted in aerogels with uniform pore structures with minimal structural collapse during drying. The cross-linked aerogel’s low density led to superabsorbent and oil/water separation properties. Zhang *et al*.^21^ successfully fabricated crosslinked CNF/MFC aerogels with fast shape recovery properties in water. The resultant aerogels were robust enough to withstand a harsh solvent environment and mechanical agitation. Heath *et al*.^26^ fabricated high porosity aerogels through solvent exchange with alcohol followed by super critical CO_2_ drying, and the resultant aerogels can lead to potential applications such as supports for catalysis, filters, or storage materials. There are numerous other cellulosic aerogel fabrication approaches. Yet, one limitation is the inability to obtain aerogel 3D structures in a customizable manner to enable rapid fabrication of objects with complicated shapes, which has limited the aerogels’ applicability, as they cannot adopt to specific needs as required by various applications. As a result, there have been increasing interests in utilizing 3D printing and wood based-materials for the fabrication of cellulosics structures with complex shapes. For example, Gatenholm *et al*. reported 3D bioprinting of dissolve cellulose and functional nanofibril hydrogels with the potential for conductive electronic and other industrial applications^27, 28^. Hart *et al*. reported 3D printing of cellulose acetate based structures that may be suited for medical or surgical use^29^. Studart *et al*. have reported CNC architectures printed via the DIW approach, where CNC alignments were achieved during 3D printing^30^. In this paper, we report DIW printing of pure CNC aerogels with complex structures and customizable inner pore architectures. The unique dual pore CNC aerogels structures could potentially have the efficient cell integration capability as needed in tissue engineering applications. To ensure that the ink can be extruded efficiently during deposition and maintain overall 3D structures after deposition, various high concentration CNC gels with shear thinning behavior were prepared to test their 3D printability. To further enable inner pore architecture and overall structural customization, it was necessary to carefully control the aerogel’s Computer Aided Designs (CAD), gel formulations, DIW processing parameters, and nozzle tip size. Ultimately, this work provided a unique solution for fabricating dual pore CNC aerogel 3D structures with controllable porosity, resolution, overall shape, and inner pore architecture.

## Results

### CNC hydrogel ink characteristics

**Figure 1:**
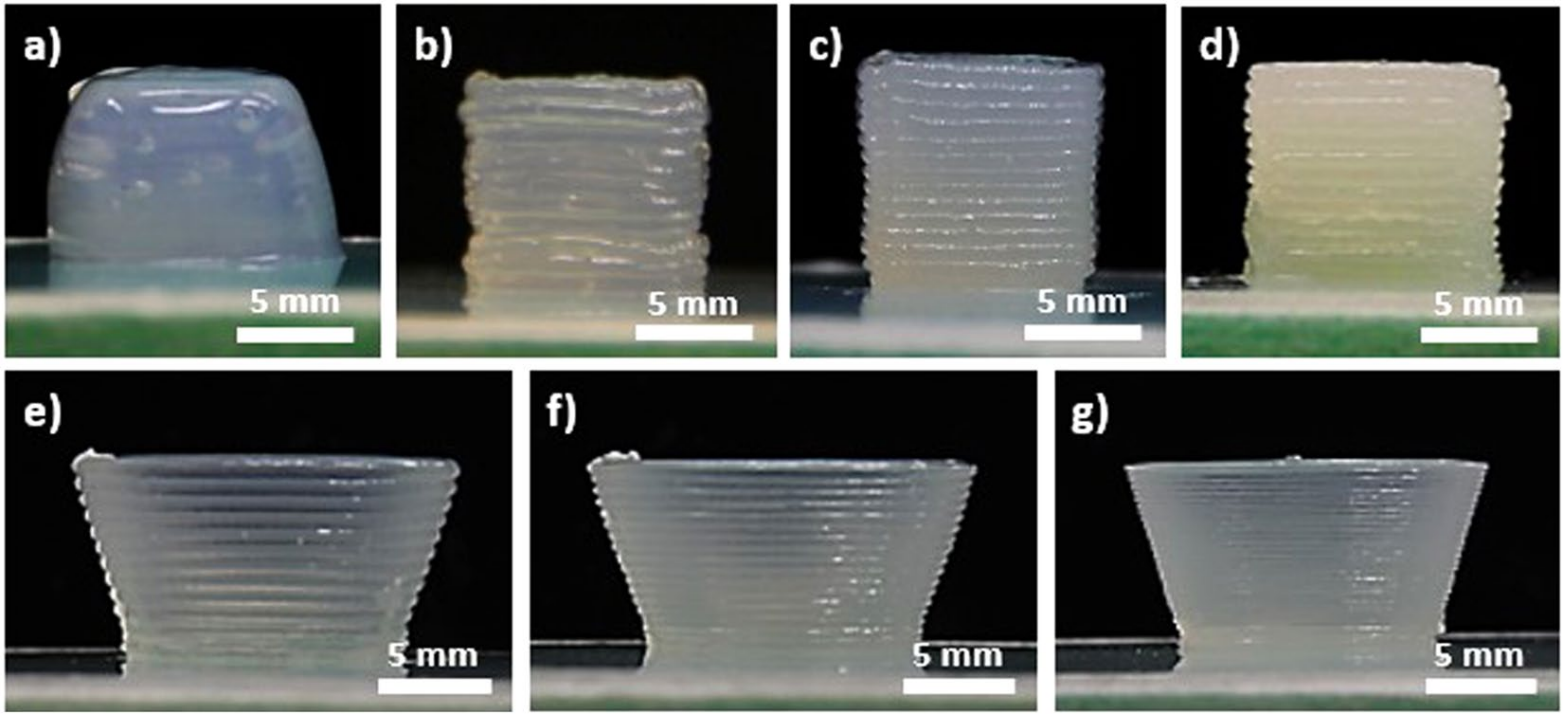
From 500 µm nozzle tip size, 1 cm^3^ cubic CNC gel structures at (**a**) 11.8, (**b**) 15, (**c**) 20, and (**d**) 30 wt% are illustrated. Using 20 wt% CNC gel, bowl structures from nozzle tip size of (**e)** 500, (**f**) 400, and (**g**) 200 µm are also illustrated.

CNC gels at 11.8, 15, 20, and 30 wt% in water were prepared and 3D printed into a simple 1 cm^3^ cubic structure using the DIW approach (Fig. 1a–d). After freeze drying, the aerogels’ density and porosity were measured from their weight and volume. CNC gels with weight percent between 11.8 and 30% resulted in an aerogel density range of 127 to 399 mg/cm^3^, while porosity ranged from 92.1 to 75.0%. As the weight percent of CNC increased, the density increased while the porosity decreased linearly. Both density and porosity can be extrapolated to the theoretical bulk cellulose’s density and porosity of 1600 mg/cm^3^ and 0%, respectively (Fig. S1 and Table S1 in Supplementary Info). The linear decrease in porosity can be explained by decreasing pore sizes within the aerogel. During freeze drying, smaller ice crystals were generated in structures with higher CNC concentrations due to decreasing water content in the gel. This is supported by SEM analysis of cross-sections from aerogels with different CNC concentrations, where decreasing macropore size was observed as the concentration of CNC increased (Fig. S2 in Supplementary Info). The formation of macroporous structures was consistent with other literature reports on water based freeze drying of cellulosic aerogels^31, 32^. At low freezing rates, ice crystal growth was much more dominant than ice crystal nucleation. As ice crystals grew, they further expanded and forced CNC to aggregate in addition to CNC’s original tendency to form strong hydrogen bonds with themselves.

The viscosities of CNC gel were also evaluated through cone and plate viscometer tests, and the viscosity was found to increase with CNC concentration (Fig. S3 in Supplementary Info). Furthermore, the CNC gel changed from shear thickening to shear thinning as CNC weight percent increased. This was confirmed by the change in concavity of the shear stress versus shear rate curve as the CNC weight percent increased, which suggested that high shear can induce some degree of CNC alignment, as shear rate and CNC concentration increased (Fig. S4 in Supplementary Info). In effect, the shear thinning effects for efficient gel deposition during DIW were demonstrated. During gel extrusion, the shear thinning behavior favored efficient gel flow. After deposition, the high viscosity of the gel allowed the printed features to be maintained. Based on the viscosity of the gel, different pressures were required for gel deposition. For each CNC gel concentration and nozzle tip size combination, the width and height of the gel filament during printing can be controlled by optimizing the deposition pressure, printing speed, G-code extrusion width, and G-code layer height. By optimizing these processing parameters, targeted gel filament dimensions and CNC gel structures with high structural and dimensional accuracy can be reproducibly printed. The viscosity, corresponding pressure needed for deposition, the mean absolute deviation, and the mean smoothness deviation from gel structures with different CNC concentrations are summarized in Table S2 in the Supplementary section. CNC concentrations higher than 30 wt% were not printed, as the power of our probe homogenizer to adequately homogenize the CNC gel was limited, and the pressure required for deposition was higher than the maximum pressure that could be controlled by our available air pressure controller. Based on edges from the printed structures, the mean absolute deviations and mean smoothness deviation were also provided to evaluate the printed structure’s shape fidelity and edge smoothness, respectively. Mean absolute deviation determines how the edge deviates from a straight line, and mean smoothness deviation determines how the edge deviates from a best fitted line through the edge. A lower mean absolute deviation represents structures with higher shape fidelity as these structures produce straighter edges, and a lower mean smoothness deviation represents structures with smoother edges.

### Evaluation of shape fidelity, edge smoothness, and overall print quality of DIW printed CNC hydrogel structures

Due to the increase in viscosity, the pressure needed to deposit the gel increased as the weight percent of CNC increased. The mean absolute deviation also decreased as CNC weight percent increased, which suggested that higher viscosity gel can better maintain the printed edges and overall structure. The mean smoothness deviations were maintained at low values across all CNC concentrations, which suggested that there was good continuity along the edge even though the edge was composed of individually deposited layers. Printed structures from 11.8 wt% CNC could retain overall shape right after printing, but the relatively low viscosity gel usually resulted in structural sagging within 5 to 10 minutes (Fig. 1a). As a result, the mean absolute deviation from 11.8 wt% CNC gel was significantly higher than the other CNC concentrations. Yet, the sagged structure still had a highly smoothed edge, owing to the CNC gel’s liquid gel-like feature at 11.8 wt%. This led to a relatively low mean smoothness deviation, as the edge was highly smooth. Ultimately, the print quality was improved as the viscosity and CNC concentration of the gel increased.

To further improve the printing quality, the 500 µm nozzle tip was replaced with nozzle tip sizes of 400 µm and 200 µm. Using 20 wt% CNC gel, bowl structures were printed from different nozzle tip sizes (Figs 1e–g and S6 in Supplementary Info). To quantitatively assess the effect of nozzle tip sizes on print quality, 1 cm^3^ cubic-shaped gel structures were again printed using nozzle tips of different sizes (500, 400 and 200 µm). Then, the mean absolute deviation and the mean smoothness deviation were evaluated. The results are summarized in Fig. S7 and Table S3 in the Supplementary Info. Overall, the pressure required for deposition increased as the nozzle tip size decreased. The straightness and smoothness of various prints were also found to be similar across a variety of nozzle sizes. This suggests that the deposition pressure, printing speed, G-code extrusion width, and G-code layer height can be optimized for each nozzle tip size in a way that achieves prints with similar edge quality. Nevertheless, even though the overall edge straightness and smoothness were similar for all nozzle tip sizes, the 200 μm resolution print still had the best printing quality and surface smoothness based on qualitative visual inspection. As the nozzle tip size decreased, the interconnectivity of each layer from the z-directional surface became more continuously smoothed. Overall, the print quality and surface smoothness improved as the printing resolution increased. However, the time required to complete the print also increased as the nozzle tip resolution increased. In fact, the estimated printing completion time increased from 20 mins/cm^3^ to 130 mins/cm^3^ of ink when the printing resolution increased from 500 µm to 200 µm. As a result, there was an inherent tradeoff between the printing resolution and the time required to complete the print.

### Customizable CNC aerogel structures, their mechanical properties, and dual pore scaffolds with tailorable inner pore architectures

**Figure 2:**
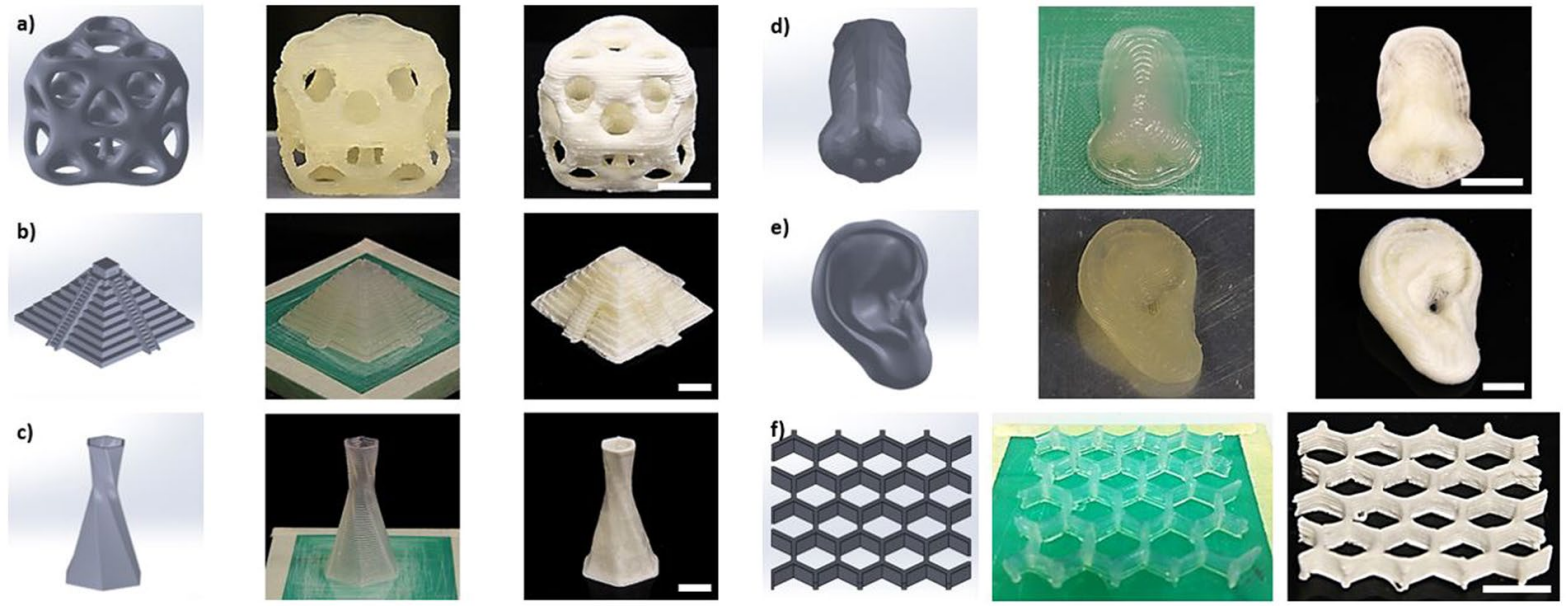
DIW 3D printed (**a**) octet cube, (**b**) pyramid, (**c**) hexagonally twisting vase, (**d**) nose model, (**e**) ear model, and (**f**) honeycomb from 20 wt% CNC gel and 500 µm nozzle tip. First column is CAD model, second column is DIW 3D printed gel structures, and third column is resultant structures after freeze drying. Displayed scale bars are 1 cm.

To demonstrate the flexibility that can be realized by controlling the aerogel’s 3D structures, 20 wt% CNC gel was printed into various structures, such as octet cube, pyramid, hexagonally twisting vase, nose model, ear model, and honeycomb with a nozzle tip size of 500 μm. The resultant gel and freeze dried aerogel structures are illustrated (Fig. 2). It is evident from Fig. 2 that the printed gel structures closely resemble the CAD designed structures, thus demonstrating the ability to print CNC gel structures directly from CADs. Furthermore, there was no observable aerogel shrinkage or damage after freeze drying. The high Young’s modulus and strong hydrogen bonding potential provided by CNC ended up limiting structural collapse or shrinkage during the ice crystal sublimation process. It should also be noted that gel structures were successfully printed without any support materials, which significantly reduced waste material. This was likely due to the CNC’s inherent light weight, and high viscosity at 20 wt% CNCs.

**Figure 3:**
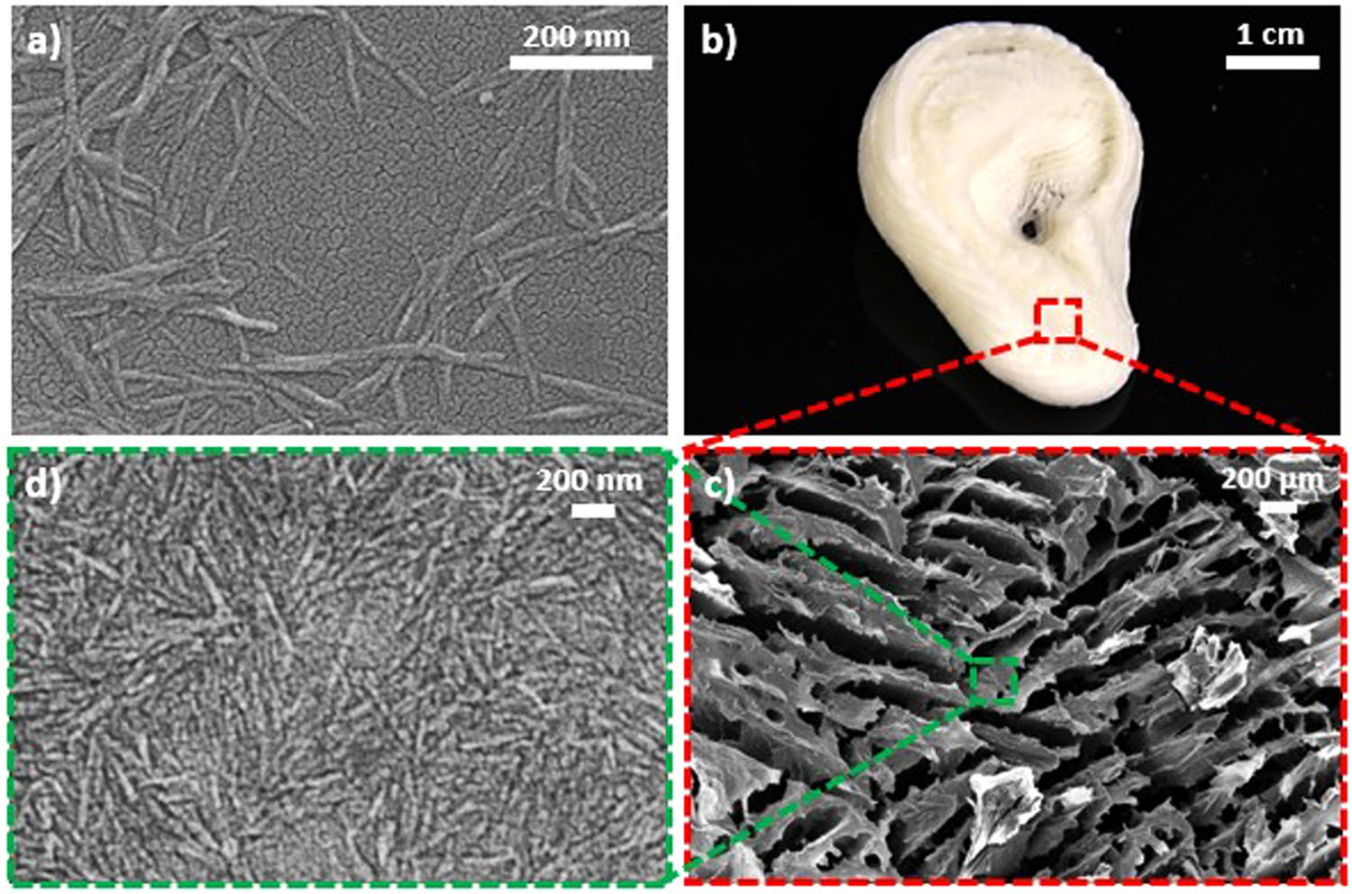
(**a**) SEM images of CNCs used during hydrogel generation for DIW processing. (**b**) After DIW 3D printing followed by freeze drying, an ear model without needing support material was successfully fabricated using 20 wt% CNC gel and 500 µm nozzle tip. (**c**) Macroporous structure of the aerogel was confirmed by SEM analysis. (**d**) CNC, in a highly closed packed manner, that constituted the aerogel structure are also illustrated.

The CNCs used in this study were characterized by SEM (Fig. 3a), which illustrated a CNC length and width of approximately 400 nm and 30 nm, respectively. After DIW 3D printing and freeze drying, the free-standing ear model aerogel structure (Figs 3b and S8 in Supplementary Info) was used to characterize the internal surface morphology through SEM. As seen in the aerogel’s SEM image (Fig. 3c), the porous nature of the CNC aerogel was confirmed. A distribution of macropore sizes, between 20 µm and 800 µm, was observed (Fig. S9 in Supplementary Info). Upon higher magnification, the high concentration CNCs that constituted the aerogel structure were also observable (Fig. 3d). It was found that CNCs were in highly close packed manner when compared to Fig. 3a, which illustrated the strong self-hydrogen bonding potential of CNCs after freeze drying. Interestingly, the actual pore morphology in Fig. 3c appeared to be elongated into platelet like shapes. Furthermore, nearby pores seemed to align in parallel with one another. This type of pore morphology is typically observed in aerogel processed at steady and low freezing rate^20, 31^, which was the condition used in this work. More specifically, the physics of water freezing seem to favor an anisotropic ice crystal growth that are parallel to the movement of the ice-water freezing front. After sublimation, the resultant lamellar microstructures resembled the shape of platelets that are aligned parallel to one another. Depending on water content, freezing approach, and cooling speed, the freezing front could be controlled to tailor the thickness of the platelets^33, 34^. Overall, this analysis demonstrated that it was possible to obtain porous aerogels with well controlled shapes from purely cellulosic materials by using the DIW technique followed by freeze drying. These structurally complex CNC aerogels with controllable porosity promise great potential for tissue engineering applications due to their open cell type porous structures. This can allow solvent absorption, cell seeding medium infusion, oxygen permeation, nutrient transport, cell growth, or metabolic waste removal to occur within the aerogel. A macropore size distribution of 20 to 800 µm may also allow effective cell growth due to the comparable cell spreading length and pore sizes^35, 36^. In an ideal scenario, cell growth can migrate through the aerogel using the smaller to medium pore sizes. Meanwhile, the larger pore sizes in the distribution can enable nutrient and metabolic waste diffusion to occur simultaneously until complete cell migration is achieved^37^. In fact, the compatibility between cellulose based materials and bone or cartilage tissue has already been demonstrated^38–41^, where cell integration and growth onto cellulose-based aerogels was successful. We have also tested cellulosic aerogels as culture scaffolds previously, where 3T3 NIH cells were compatibilized with the cellulosic-scaffolds^42^.

The mechanical properties of the CNC aerogels were also characterized by MTS compression analysis (Fig. S10 in Supplementary Info). The initial Young’s modulus was determined to be approximately 7 MPa. The stress and strain before the first crack formation were 0.133 MPa and 6.65%, respectively. The CNC aerogel’s high stiffness but brittle nature was evident, which could lead to structure collapse during cell incorporation and growth. Since the CNC aerogel structures were not cross-linked, structural dissolution upon immersion into water or other organic solvents was also possible. Nevertheless, cross-linking of CNC aerogels structures were achievable by incorporation of a wet-strength additive known as, polyamide-epichlorohydrin (Kymene). Even through a small amount of Kymene addition to the CNC aerogel (2.5 wt% Kymene), the mechanical properties of the CNC aerogel were improved in both air and water environment. After cross-linking, the Young’s modulus increased from 7 MPa to 8.94 MPa. The CNC aerogel also became less brittle, which led to a stress-strain curve with a densification region. The stress and strain before densification were determined to be 21.5 MPa and 77.9%, respectively. While the CNC aerogel was immersed in water, the corresponding wet state mechanical properties were similarly evaluated. The resultant Young’s modulus was determined to be approximately 380 kPa, while the stress and strain before the first crack formation were 0.137 MPa and 30.3%, respectively. It appeared that water absorption had significantly softened the aerogel due to hydrogen bond disruption, but the compressibility of the water saturated CNC aerogel still improved from 6.65% to 30.3% through Kymene cross-linking. By cross-linking the CNC aerogel, cell growth without structural collapse may be possible.

**Figure 4:**
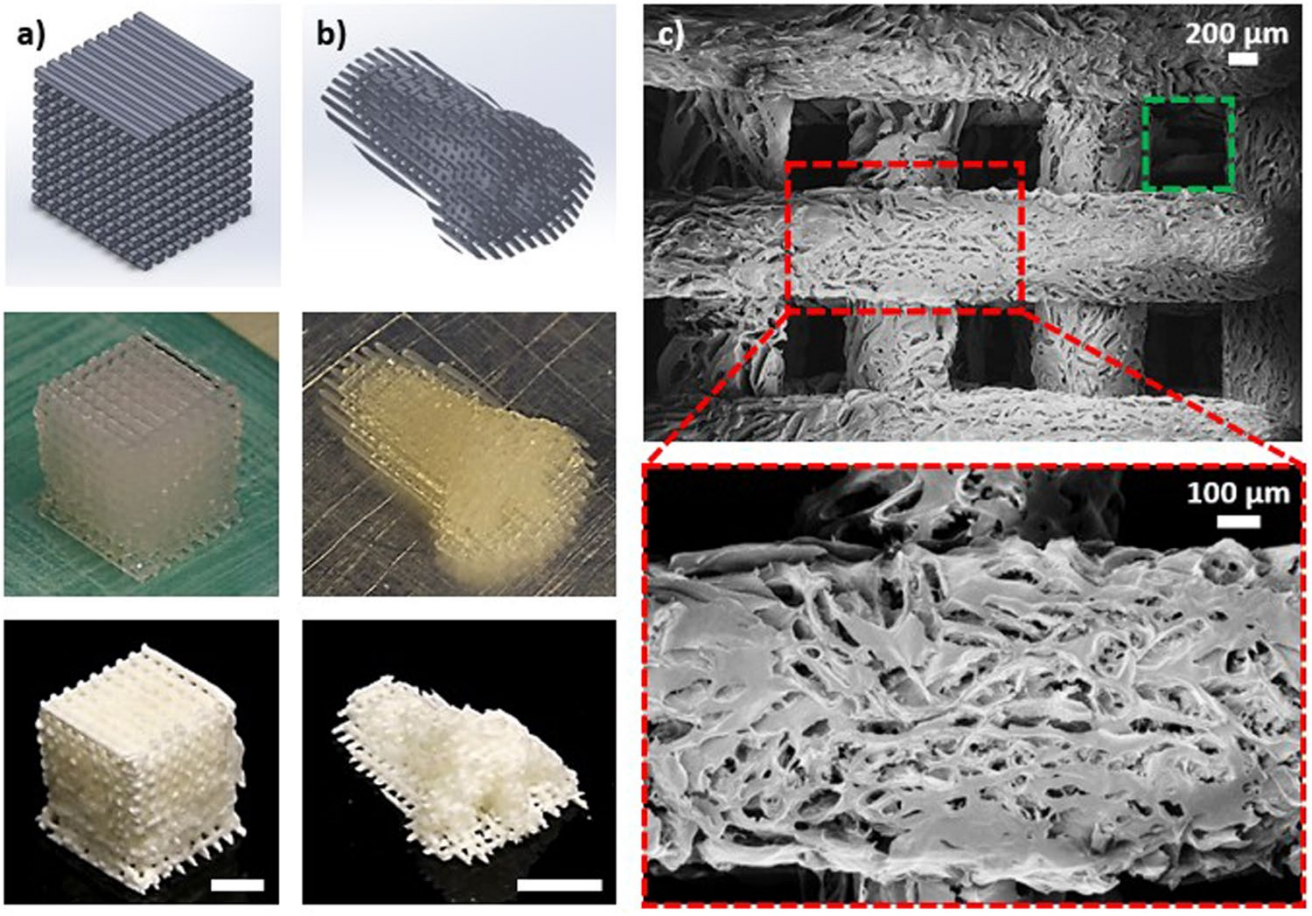
DIW 3D printed dual pore scaffold aerogel in a 0–90° configuration for (**a**) a cube and (**b**) a nose model. (**c**) The corresponding SEM images illustrate an average structural pore size of about 600 µm (green box) and a random pore size distribution of 20 to 800 µm (red box). 20 wt% CNC gel and 500 µm nozzle tip was used. Unless specified, displayed scale bars are 1 cm.

To enable more efficient tissue regeneration onto scaffolds, recent efforts have been focused on fabricating dual pore structures with both structural pores and random pores. Dual pore scaffold systems had an advantage over traditional fully random porous structures for facilitating cell growth because the larger structural pores could enhance nutrient and oxygen transport, and this promoted homogenous cell proliferation throughout the entire scaffold. Meanwhile, the smaller random pores provided the large surface areas necessary for high density cell growth. In fact, several cell-scaffold integration studies have already confirmed that the dual pore scaffold system led to improvements in cell count, metabolic activity, and uniformity of cell distribution when compared to fully random porous structures^43–45^. To further demonstrate the potential to utilize DIW based 3D printing to fabricate biocompatible CNC aerogel scaffolds for tissue engineering applications, various dual pore CNC aerogel scaffolds were fabricated. More specifically, filaments were fabricated with orientation configurations of 0–45°, 0–90° (Fig. 4), and 0–45–90–135° throughout each z-directional layer in the 3D gel structures (Fig. S11 in Supplementary Info). After freeze drying, porosity measurements indicated that the porosity can be tuned from about 80% to 90% purely from the filaments’ orientational designs when using 20 wt% CNC gel. As a demonstration, a nose model was also fabricated in a 0–90° structural configuration (Fig. 4b). The G-code extrusion width and height was 700 µm, while the filament spacing was specified to be also 700 µm. From SEM analysis, the filaments had an average width of about 750 µm (Fig. 4c). The increase in filament width could be attributed to a small degree of gel spreading after the deposition. Due to the slight increase in filament width, an average structural pore size of about 600 µm (green box) was observed. Moreover, a distribution of macropores (red box) was again observable on individual CNC filaments. Overall, the ability to utilize the DIW and freeze drying technique to fabricate pure CNC dual pore aerogel 3D structures was demonstrated. Based on the type of cells and their environmental growth requirements, the necessary structural and inner pore architectures can be controlled by tailoring the aerogel’s CAD architectural designs, DIW processing specifications, gel formulations, and nozzle tip size. Overall, highly customizable CNC aerogel structures were successfully printed. Cell integration onto these aerogels and further improvements on their wet state mechanical properties will be the focuses of future investigations.

## Discussion

A method that can 3D print pure cellulose nanocrystal aerogels with well controlled overall structures and dual porous structures was developed. Gels at different weight percent of CNC were prepared and printed into complex 3D structures through the DIW approach. After freeze dry processing, an aerogel density range of 127 to 399 mg/cm^3^ and a porosity range of 92.1 to 75.0% were achievable with CNC gel concentrations of 11.8 to 30 wt%. Print quality assessments suggested that the increasing weight percent of CNC could provide straighter and smoother edges, while the surface smoothness could be improved by using a smaller nozzle tip size albeit at the cost of longer printing time. DIW 3D printed structures were also maintained with minimal shrinkage or damage even after freeze drying. Various dual pore CNC aerogel scaffolds were also successfully processed, which promised great potential for a high degree of structural and inner pore architecture customization for different tissue engineering applications. These results demonstrated the highly capable 3D printing and freeze drying approach for creating complex and customizable aerogels suited for target application requirements.

## Methods

### Sample Preparation

Freeze dried CNCs derived from wood pulp and 11.8 wt% CNCs in water suspension were purchased from University of Maine Process Development Center (Orono, ME, USA). Both the freeze dried CNCs and the 11.8 wt% CNC suspension were used as received. Wet-strength additive cross-linker, polyamide-epichlorohydrin (Kymene), was kindly given by Ashland Hercules Incorporated(Covington, KY, USA) and used as received. Water was used in all experiments. Freeze dried CNCs based on targeted weight percent was measured by a gravimetric balance, and then mixed with water. The mixture was further homogenized by a high shear probe homogenizer (T18 basic, Ultra Turrax, IKA Works Inc., Wilmington, NC, USA) at 10,000 rpm until a homogenous gel was obtained. The homogenized CNC gel mixture was then loaded into a syringe equipped with a tapered type nozzle. Next, 3D structures designed from CAD design software SolidWorks (Dassault Systems, SolidWorks Inc., Waltham, MA, USA) were exported into STL formats, which were then interfaced with the Repetier software (Hot-World GmbH & Co. KG, Willich in North Rhine-Westphalia, Germany) to generate G-codes for each layer in the 3D structures. The G-code designated the nozzle (x, z motion) and stage movement (y motion) for 3D structure construction through a layer by layer fashion. Compressed air was generated by a 135 psi and directed to an air pressure controller (Ultimus V, Nordson EFD, East Providence, RI, USA), where a specified pressure was used to deposit the CNC gel through the nozzle. With a nozzle movement speed of 2 mm/s, 3D CNC gel structures were printed and then placed in a −20 °C freezer overnight. A lyophilizer (VirTis Freezemobile 25EL Sentry 2.0, USA) operating under 20 mTorr vacuum was then used to sublime the frozen ice crystals for a duration of 2 days before 3D aerogel structures were obtained. For cross-linkable CNC aerogel, a trace amount of Kymene (2 wt% based on dry cellulose mass) was added to the CNC gel and then homogenized by a high shear probe homogenizer. After DIW and freeze drying, the aerogel was cross-linked in an oven at 120 °C for 3 hours. Then, the cross-linked CNC aerogel was immersed into a 2.5 wt% Kymene in water solution for 3 hours before the Kymene infused CNC aerogel was removed from solution for another freeze drying and oven treatment cycle.

### Density Measurement and Porosity Calculation

A 1 cm^3^ cubic structure was printed from the CNC gel mixture. After freeze dry processing, the mass and volume of the aerogel was measured by a gravimetric balance and a caliper, respectively. After the density of the aerogel was measured, the porosity of the aerogel (Φ) was determined by^22^.

1 $${\rm{\Phi }}=(1-\frac{{\rho }_{aerogel}}{{\rho }_{s}})\ast 100 \% $$

where *ρ*_*aerogel*_ is the calculated density of the aerogel sample, and *ρ*_*s*_ is the bulk density of the aerogel. The bulk density of the aerogel was set as the density of cellulose, which is 1600 mg cm^−3^ based on previous literature reports^7, 11^.

### FE-SEM Characteriation

To prepare the sample for SEM, a drop of diluted CNCs in water was deposited onto a thin glass slide. Then, the glass slide was attached to the SEM sample holder with conductive double sided tape. After the water was evaporated under ambient air, the sample was sputter coated with gold for 60 seconds at 20 mA current with a Quorum Q-150T ES Sputter to help prevent charging. Planar cross-sections from CNC aerogel structures were also obtained through cryofracture using liquid N_2_; then cross-sections were again attached to SEM sample holders with conductive double sided tape and sputter coated with gold before SEM was performed at 10 kV accelerating voltage.

### Print Quality Evaluation

The quality of the DIW printed structures was determined from analyzing the edge of a 1 cm^3^ cubic structure. First, optical images were converted into black and white images using a customized Matlab script. Then, the edge profiles from the right side of the cubes were traced into curves (Fig. S5 in Supplementary Info). From these curves, the mean absolute deviations and the mean smoothness deviations were determined according to equation (2) and (3).

2 $$Mean\,absolute\,deviation=\frac{1}{n}(\sum _{i=1}^{n}abs({L}_{i}-{L}_{avg}))$$

3 $$Mean\,smoothness\,deviation=\frac{1}{n}(\sum _{i=1}^{n}abs({L}_{i}-{L}_{i,bestfitline}))$$

where *n* is number of measured lengths, and *L* is the length determined from the Matlab script. Mean absolute deviation determines how the curve deviates from a perfectly straight line, where a lower deviation value indicates a straighter edge. On the other hand, mean smoothness deviation determines how the curve deviates from the best fitted line through the curve, where a lower deviation value indicates a smoother edge. After Matlab processing, mean absolute deviation and mean smoothness deviation in units of pixel dimensions were converted into units of millimeters. The conversion was done based on a pixel to length conversion factor, which was determined through imageJ analysis.

### Data Availability

The datasets generated during and/or analyzed during the current study are available from the corresponding author on reasonable request.

## Electronic supplementary material

Supplementary Information
