# Supplementary material for: Direct Ink Write (DIW) 3D Printed Cellulose Nanocrystal Aerogel Structures
Source: Sci Rep. 2017 Aug 14;7:8018. doi: 10.1038/s41598-017-07771-y (PMC5556020; doi:10.1038/s41598-017-07771-y)
Supplement: Supplementary file 1 — Supplementary Information [file 41598_2017_7771_MOESM1_ESM.pdf]

Direct Ink Write (DIW) 3D Printed Cellulose Nanocrystal Aerogel Structures

Vincent Chi-Fung Li, Conner K. Dunn, Zhe Zhang, Yulin Deng, H. Jerry Qi

Density and Porosity Characterization

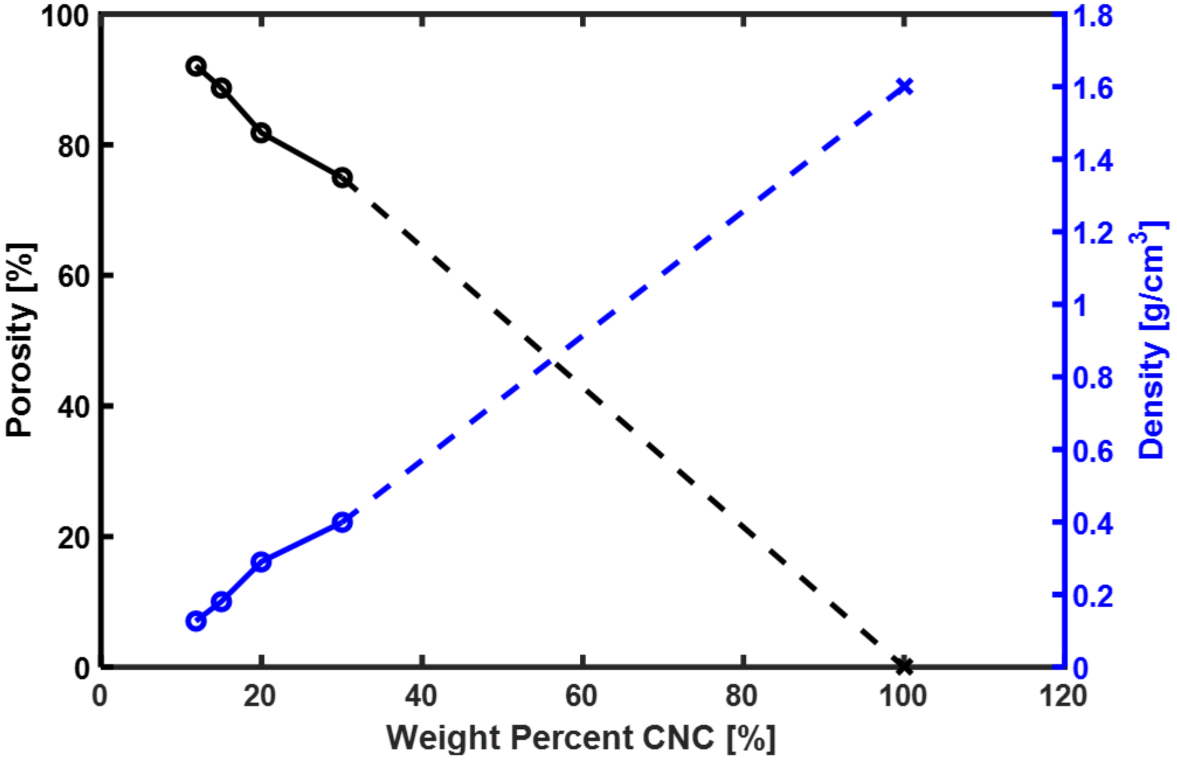

Fig. S1. Density and porosity of the resultant 1 cm^3 cubic aerogels at different CNC weight percents (Symbol O). The theoretical porosity and density for bulk cellulose is also provided (Symbol X).

Table S1. The measured density and porosity of various aerogels processed from gels with different weight percent of CNC. The theoretical porosity and density for bulk cellulose are also provided.

Weight percent CNC (%)	Porosity (%)	Density (g/cm^3)
11.8	92.1	0.127
15	88.7	0.181
20	81.8	0.291
30	75.0	0.399
100	0.0	1.600

Field Emission-Scanning Electron Microscope (FE-SEM) Characterization

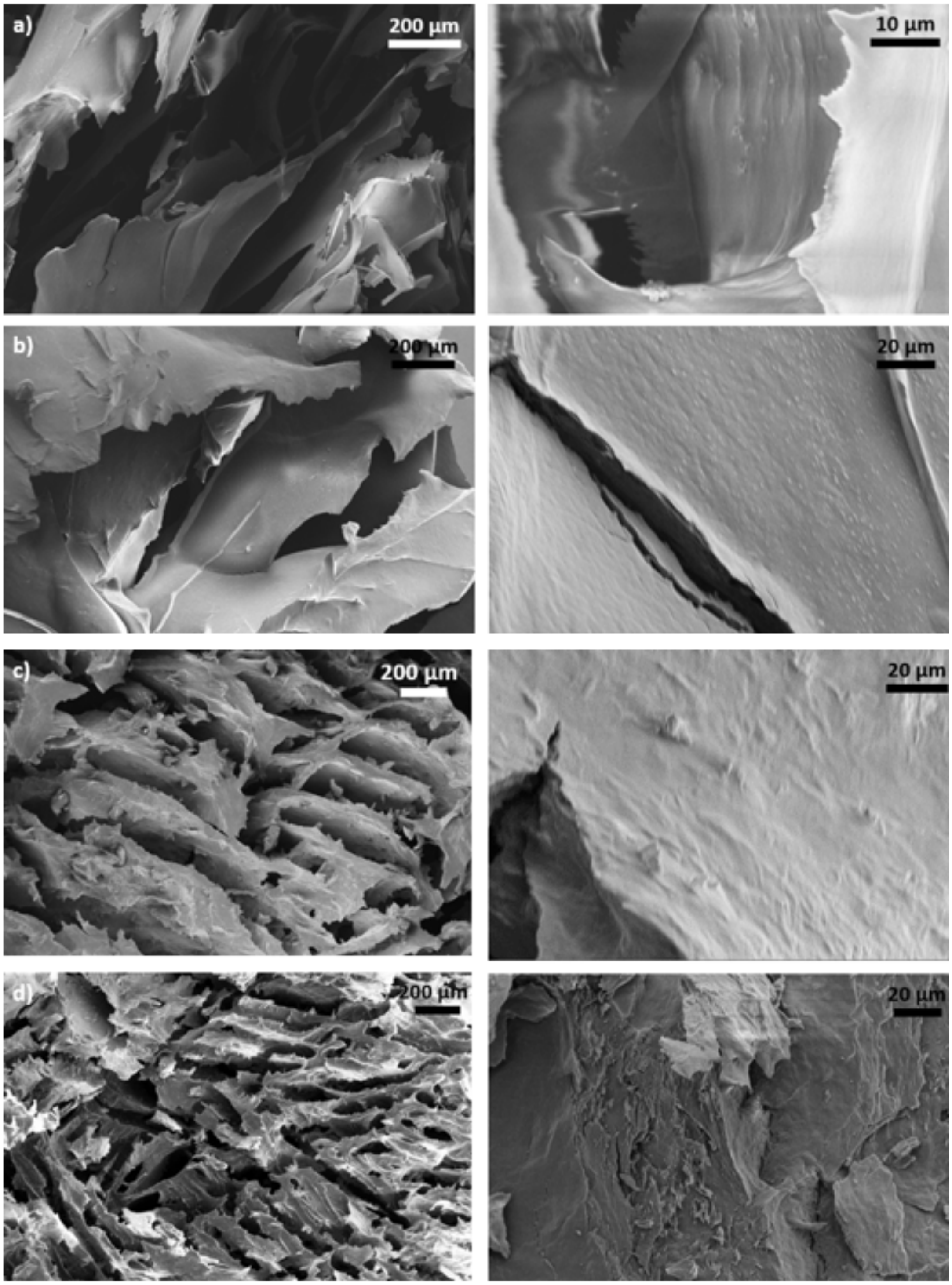

Fig. S2. Planar cross sectional SEM images of freeze dried 1 cm^3 cubic CNC aerogel structures using (a) 11.8 wt %, (b) 15 wt %, (c) 20 wt %, and (d) 30 wt % CNC gel. Cross sections were

obtained from cryofracture of DIW printed structures. Left is images taken at lower magnification, and right is corresponding images taken at higher magnification.

Cone and Plate Viscometer Characterization

The rheology of CNC gel mixtures was analyzed using the DV2TLV and DV3THB cone and plate viscometers (Brookfield Engineering Labs Inc., USA). All measurements were done at room temperature of around 22 °C, and cone spindles of CPA-41Z and CPA-52Z were used. The corresponding plates used were CPA-44YZ and CPA-44PSYZ, respectively. Viscosity was measured as a function of shear rate, where the shear rate ranged from 2 s^{-1} to 400 s^{-1}. A minimum of 10 measurements was collected for each sample at each corresponding shear rate, and averages and standard deviation values were obtained. CNC gel mixtures at 0.1 wt % were tested using the DV2TLV cone and plate viscometers with CPA-41Z cone spindle and CPA-44YZ plate. CNC gel mixtures at 11.8 wt % and higher were tested using the DV3THB cone and plate viscometers with CPA-52Z cone spindle and CPA-44PSYZ plate. The CPA-41Z cone spindle have a cone angle of 3 ° and a radius of 2.4 cm. On the other hand, the CPA-52Z cone spindle have a cone angle of 3 ° and a radius of 1.2 cm. For all viscosity characterizations, the gap separation between the cone and the plate was set at 0.013 mm. To minimize potential wall slip effects, about 3 grams of gel mixture was distributed evenly across the entire plate surface before each characterization was performed.

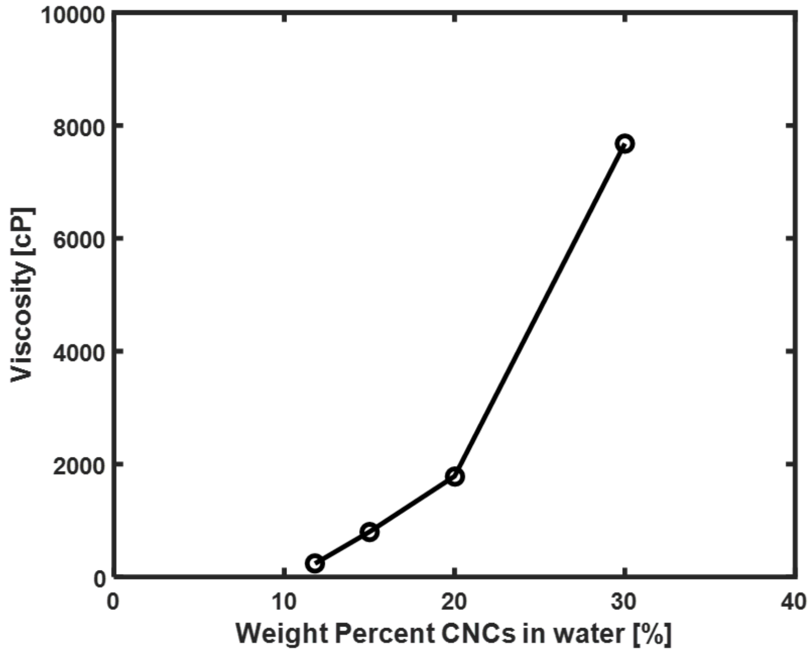

Fig. S3. The viscosity CNC gels as a function of weight percent of CNC. Viscosity displayed corresponds to the measured viscosity tested at the highest possible shear rate.

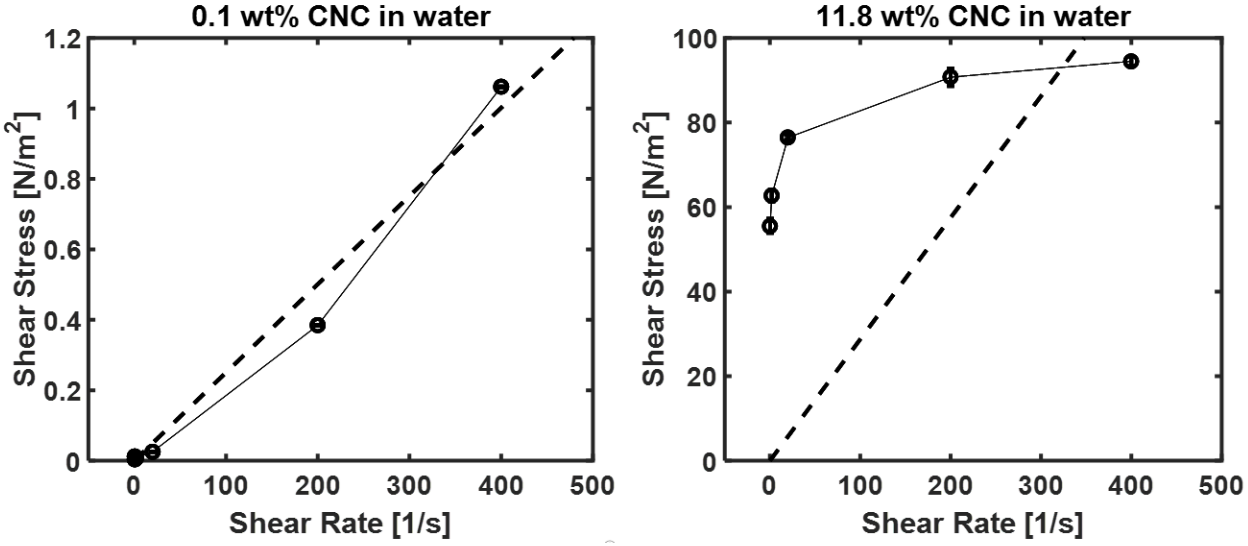

Fig. S4. The shear stress of low (left) and high weight percent (right) CNC gels as a function of shear rates. A straight dotted line was fitted through the shear stress versus shear rate curve, and the slope of the dotted line represents the viscosity of the gel if it was Newtonian. Since the shear

stress versus shear rate curve changed from concave upward to concave downward, the CNC gel changed from shear thickening to shear thinning as CNC weight percent increased.

Print Quality Based on Concentration of CNC and Resolution of Nozzle Tip

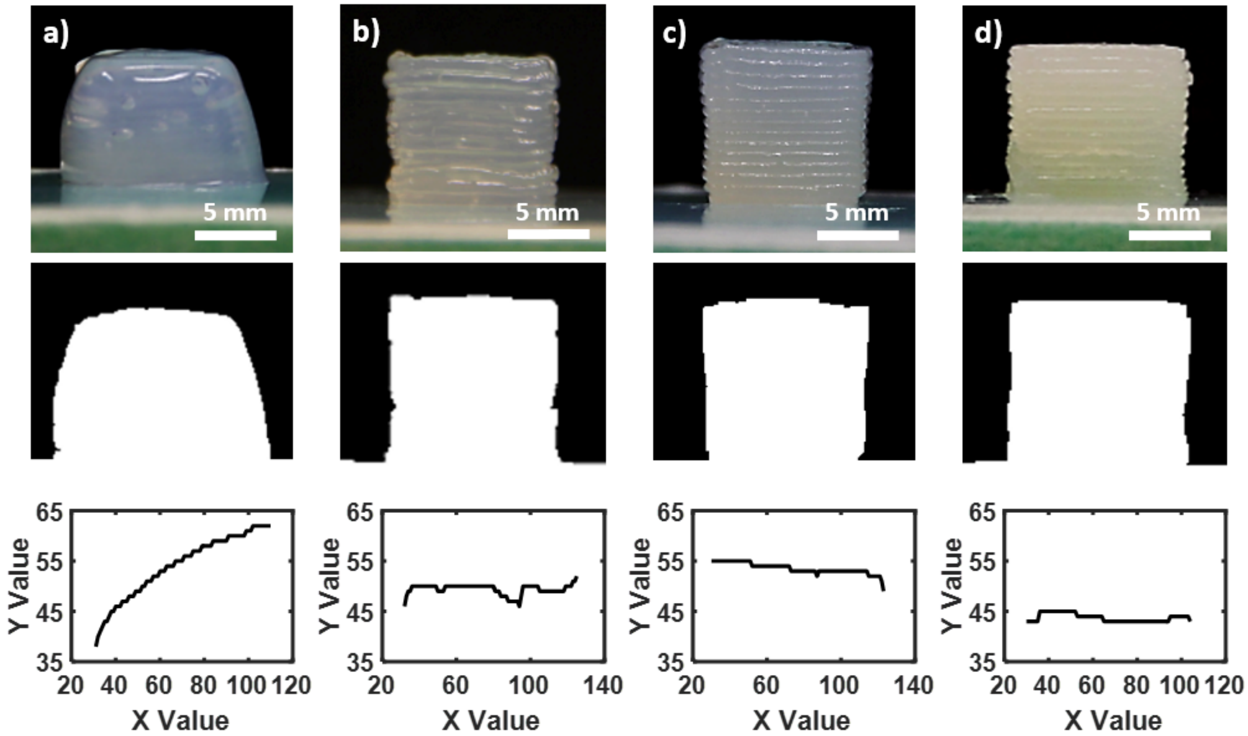

Fig. S5. Optical images (first row), converted black and white images (second row), and traced curves from the right edge of the cube (third row) for CNC gels at weight percent of (a) 11.8, (b) 15, (c) 20, and (d) 30 %. Structures were formed from a nozzle tip size of 500 μm.

Table S2. The viscosity (at highest tested shear rate), pressure applied for deposition, mean absolute deviation, and mean smoothness deviation values for different weight percent of CNC gels. G-code extrusion width of 0.9 mm, a G-code layer height of 0.7 mm, and a nozzle tip size of 500 μm were used.

Weight percent CNC (%)	Viscosity (cP)	Pressure Applied (psi)	Mean Absolute Deviation (mm)	Mean Smoothness Deviation (mm)
11.8	236.3	1	0.669	0.141
15	798.9	5	0.086	0.085
20	1785.3	20	0.083	0.040
30	7680.0	40	0.081	0.079

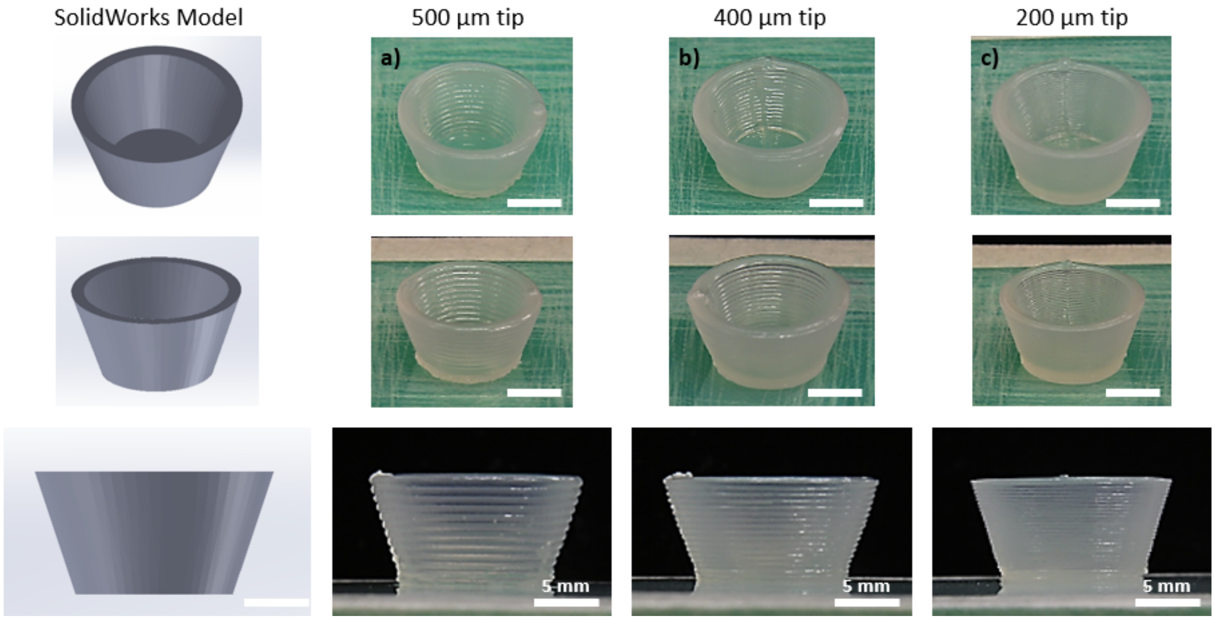

Fig. S6. DIW 3D printed bowl gel structures from nozzle tip size of (a) 500 µm, (b) 400 µm, and (c) 200 µm. Structures were formed from 20 wt % CNC. The corresponding SolidWorks model and the DIW printed bowl gel structures are illustrated from different perspectives. Unless specified, displayed scale bars are 1 cm.

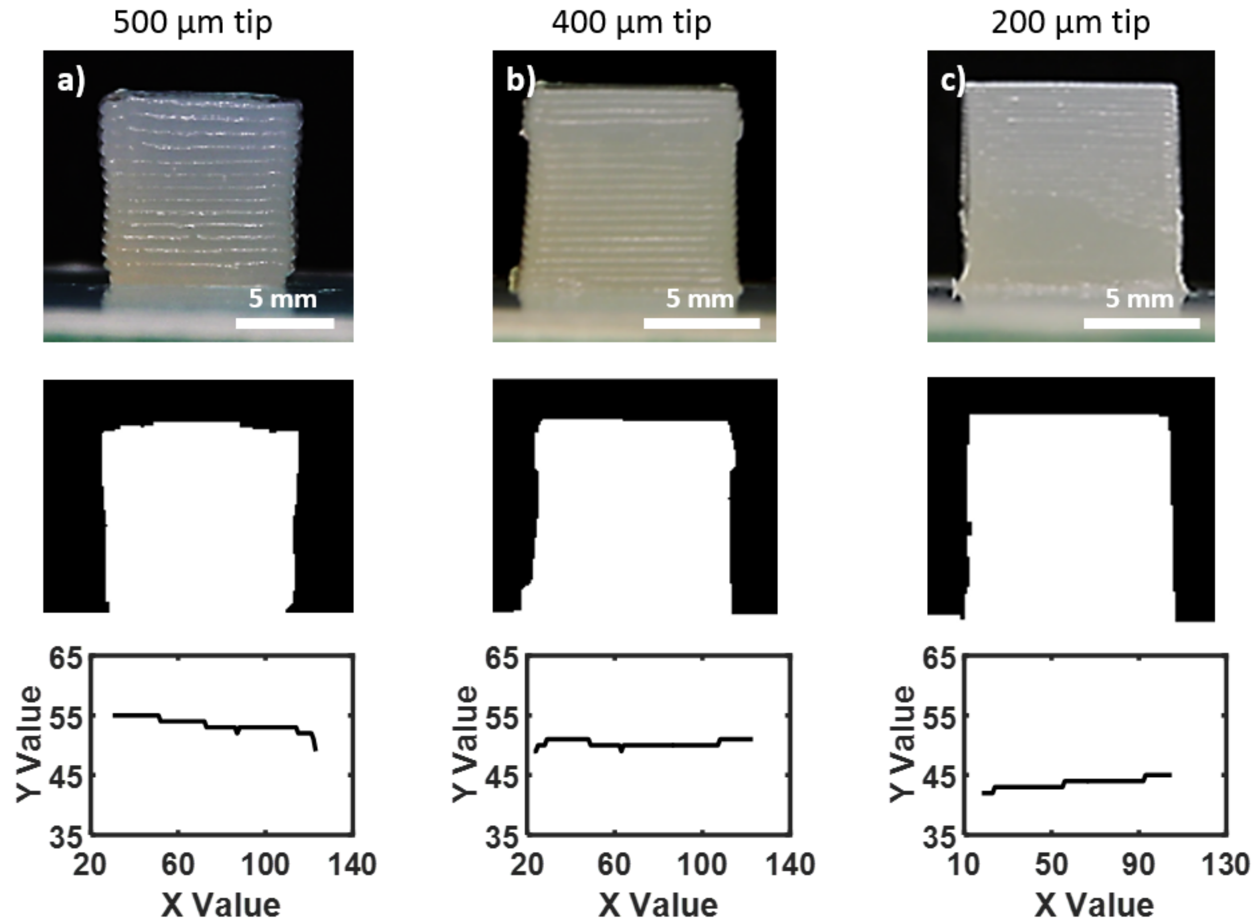

Fig. S7. DIW 3D printed 1 cm^3 cubic gel structures from nozzle tip size of (a) 500 µm, (b) 400 µm, and (c) 200 µm. Optical images (first row), converted black and white images (second row), and traced curves from the right edge of the cube (third row) for different nozzle tip sizes are illustrated. Structures were formed from 20 wt % CNC.

Table S3. The G-code extrusion width, G-code layer height, pressure applied for deposition, mean absolute deviation, and mean smoothness deviation values for different nozzle tip sizes. 20 wt % CNC gel ink were used.

Nozzle Tip Size (μm)	Width (mm)	Height (mm)	Pressure Applied (psi)	Mean Absolute Deviation (mm)	Mean Smoothness Deviation (mm)
500	0.9	0.7	20	0.083	0.040
400	0.5	0.5	40	0.038	0.033
200	0.3	0.3	60	0.079	0.030

Free Standing Ear Model Structure without Support Material

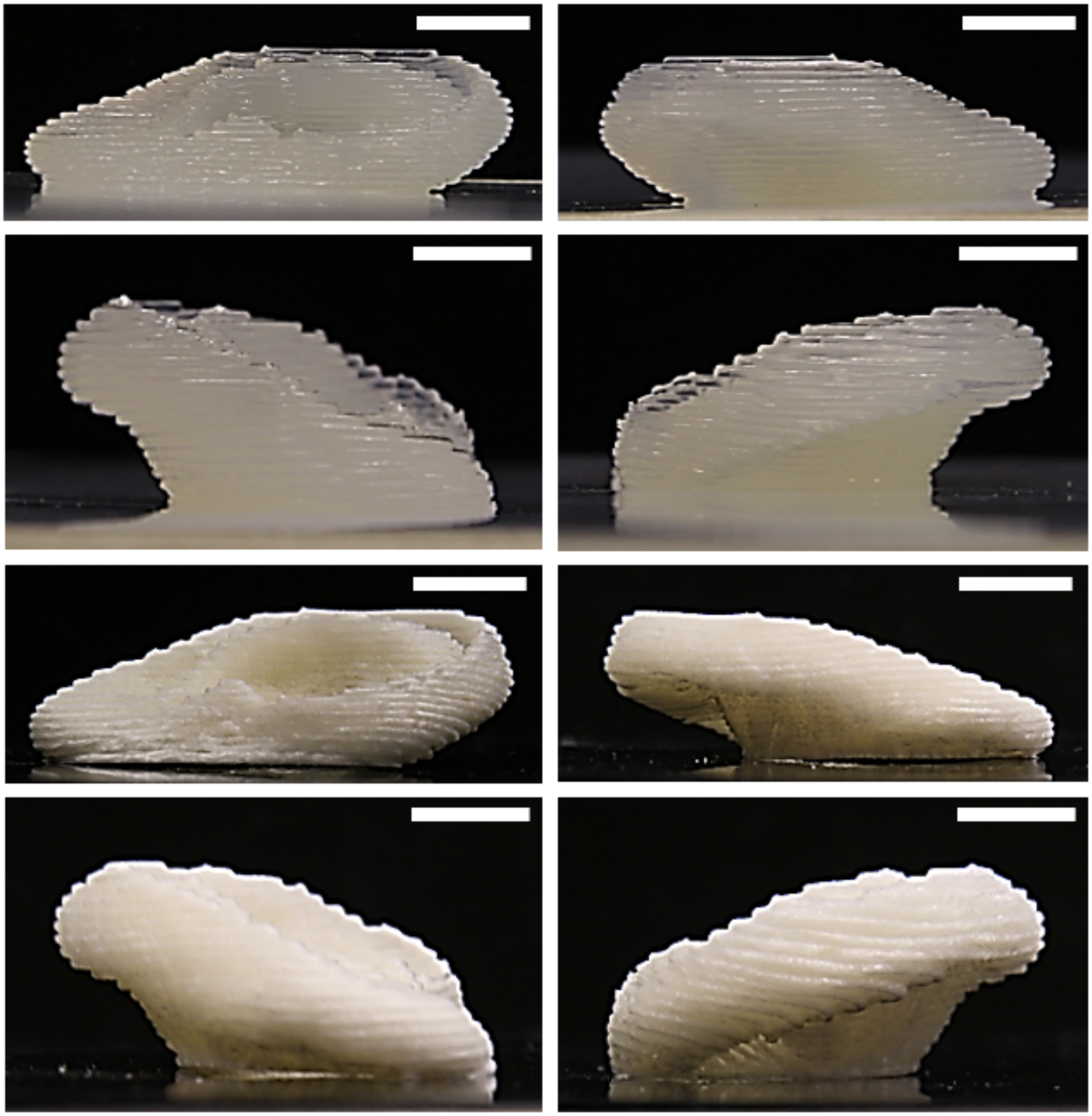

Fig. S8. Free-standing ear model as gel (Top Four) and aerogel (Bottom Four) structures without needing support materials are illustrated from different perspectives. Displayed scale bars are 1 cm.

Pore Size Distribution Analysis via SEM and ImageJ Analysis

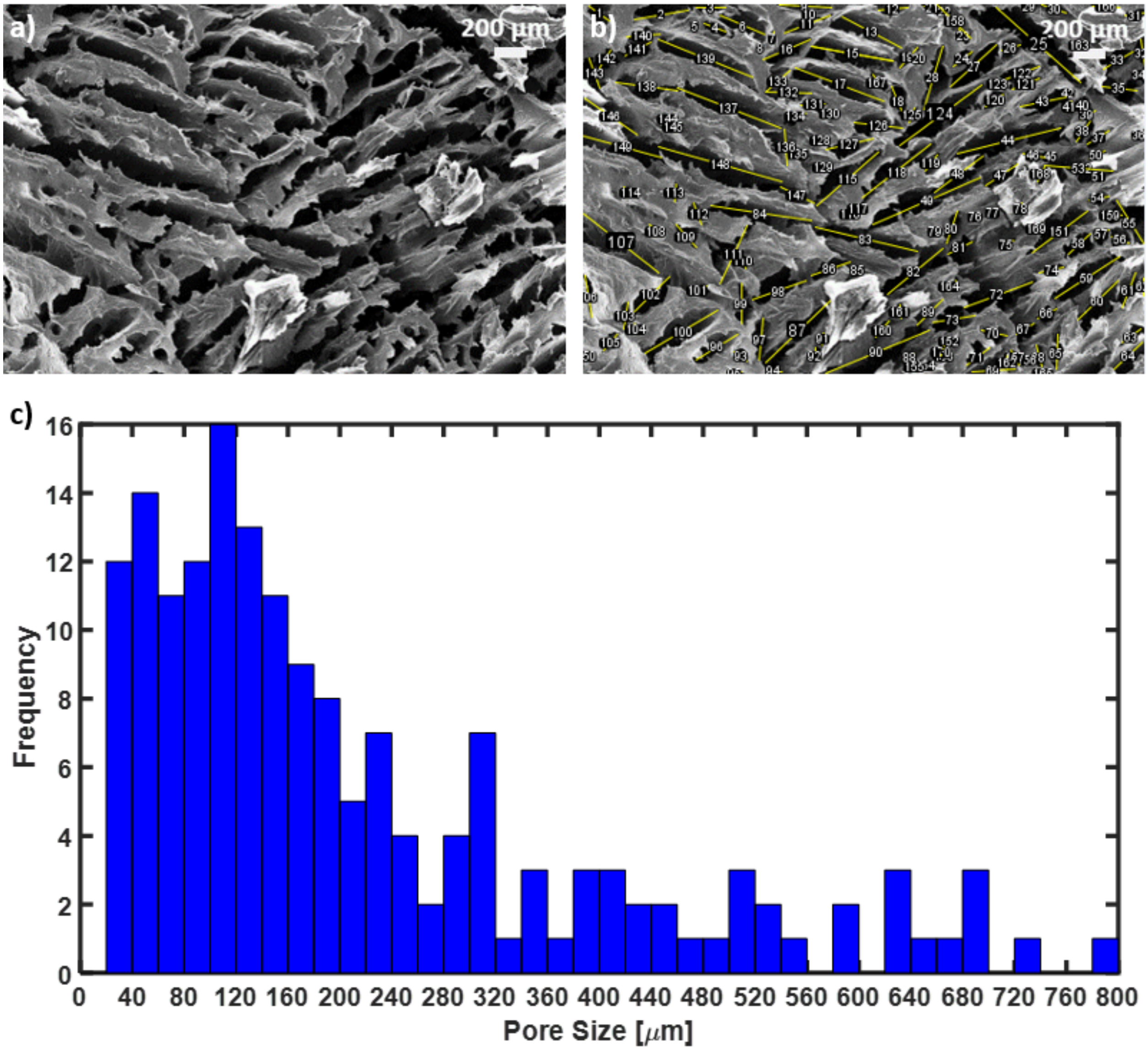

Fig. S9. (a) Resultant SEM analysis of macroporous structure in aerogel freeze dried from 20 wt% CNC gel. (b) Corresponding imageJ analysis for estimation of pore size distribution with a total pore size count of 170, and (c) the resultant pore size distribution plotted as a histogram with a bin size of 20 µm.

CNC Aerogels' Compression Mechanical Property Evaluation via MTS Analysis

1 cm^3 cubic CNC aerogel structures processed by DIW were tested by MTS (Model 312.21 with a 2.3 ton load frame) at a compression rate of 1 mm per min under ambient air and water environment. The initial Young's modulus was determine by the slope of the elastic region of the stress-strain curve. The stress and strain before break were determine at the point of first crack formation. Strain before densification was determine from the root of the best fitted line through the aerogel's densification region with an R^2 value of at least 0.98, and the corresponding stress was set as stress before densification.

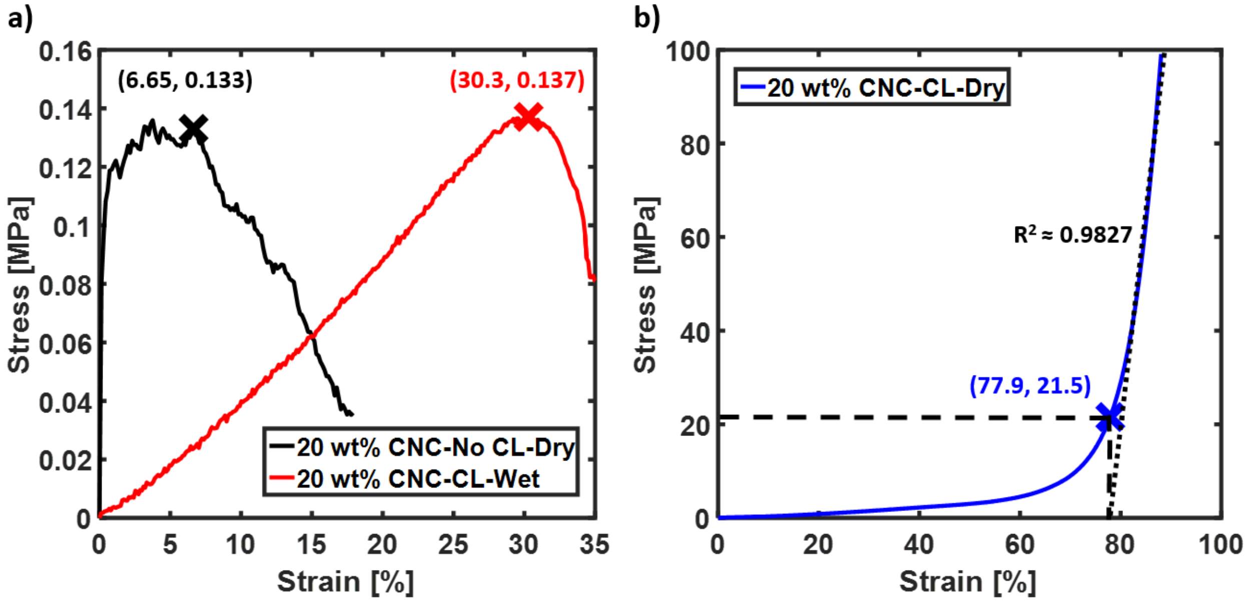

Fig. S10. (a) Resultant MTS compression curve for CNC aerogel freeze dried from 20 wt% CNC gel. The black curve represents the dry state stress-strain curve for CNC aerogel without any cross-linking (CL), and the red curve represents the wet state stress-strain curve for CNC aerogel cross-linked with 2.5 wt% Kymene. (b) The blue curve represents the dry state stress-strain curve for CNC aerogel cross-linked with 2.5 wt% Kymene. The best fitted line through the aerogel's densification region had an R^2 value of approximately 0.9827.

Scaffolds Printed at Different Filaments Orientational Configurations

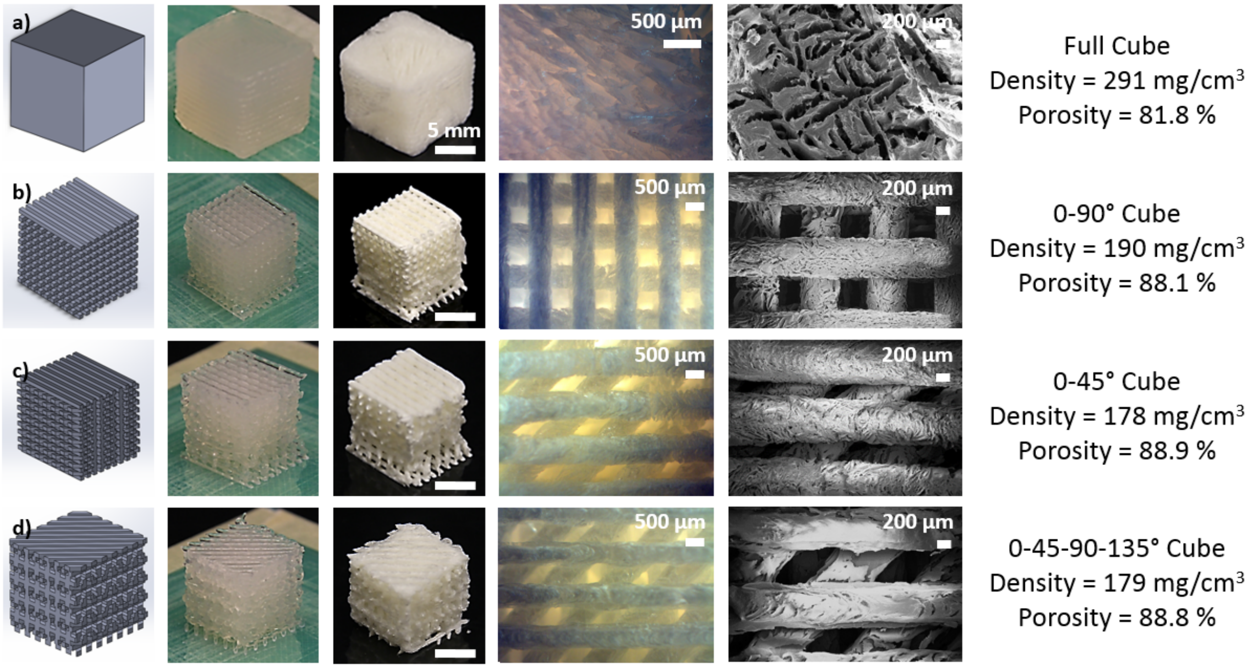

Fig. S11. (a) Fully random porous CNC aerogel scaffold and various dual pore CNC aerogel scaffolds with filaments oriented in a (b) 0-90 °, (c) 0-45 °, and (d) 0-45-90-135 ° configuration throughout each z-directional layer. The SolidWorks models, gel structures, resultant aerogel structures after freeze drying, optical microscope images, SEM images, and the measured density and porosity are displayed. Unless specified, displayed scale bars are 1 cm.
